# Cytochrome *P450 1B1 *Val432Leu polymorphism and breast cancer risk in Nigerian women: a case control study

**DOI:** 10.1186/1750-9378-4-S1-S12

**Published:** 2009-02-10

**Authors:** Michael N Okobia, Clareann H Bunker, Seymour J Garte, Joseph M Zmuda, Emmanuel R Ezeome, Stanley NC Anyanwu, Emmanuel EO Uche, Usifo Osime, Joseph Ojukwu, Lewis H Kuller, Robert E Ferrell, Emanuela Taioli

**Affiliations:** 1 Department of Epidemiology, Graduate School of Public Health, University of Pittsburgh, Pittsburgh, PA 15261, USA; 2 University of Pittsburgh Cancer Institute, University of Pittsburgh, Pittsburgh, PA 15261, USA; 3 Department of Environmental and Occupational Health, Graduate School of Public Health, and Cancer Institute, University of Pittsburgh, Pittsburgh, PA 15261, USA; 4 Department of Human Genetics, Graduate School of Public Health, University of Pittsburgh, Pittsburgh, PA 15261, USA; 5 Department of Surgery, University of Benin Teaching Hospital, Benin City, Nigeria; 6 Department of Surgery, University of Nigeria Teaching Hospital, Enugu, Nigeria; 7 Department of Surgery, Nnamdi Azikiwe University Teaching Hospital, Nnewi, Nigeria; 8 Department of Surgery, University of Port Harcourt Teaching Hospital, Port Harcourt, Nigeria; 9 Department of Epidemiology and Biostatistics, Downstate School of Public Health, State University of New York, USA

## Abstract

**Background:**

Cytochrome *P450 1B1 (CYP1B1) *is active in the metabolism of estrogens to reactive catechols and of different procarcinogens. Several studies have investigated the relationship between genetic polymorphisms of *CYP1B1 *and breast cancer risk with inconsistent results. A G → C transversion polymorphism in the heme-binding region in codon 432 of the gene results in amino acid change (Val → Leu); the Leu allele display increased catalytic efficiency for 4-hydroxylation of estradiol in some experimental systems.

**Methods:**

In this study, we utilized a polymerase chain reaction (PCR)-based restriction fragment length polymorphism (RFLP) assay to assess the relationship between this polymorphism and breast cancer risk in a case control study including 250 women with breast cancer and 250 controls from four University Teaching Hospitals in Southern Nigeria.

**Results:**

Heterozygosity for the *CYP1B1 *M1 genotype (*CYP1B1 *M1 [Val/Leu]) was associated with a significant 59% increased risk of breast cancer (OR = 1.59, 95% CI 1.01–2.58) while homozygosity for the genotype (*CYP1B1 *M1 [Leu/Leu]) conferred a non-significant 51% increased risk of breast cancer. These risk profiles were modified in subgroup analysis. In premenopausal women, harboring at least one *CYP1B1 *(Leu) allele conferred a significant two-fold increased risk of breast cancer (OR = 2.04, 95% CI 1.10–3.78). No significant association was observed in postmenopausal women (OR = 1.08, 95% CI 0.57–2.04).

**Conclusion:**

Our results suggest that the codon 432 polymorphism of the *CYP1B1 *gene is associated with increased risk of breast cancer and is particularly involved in breast cancer risk in premenopausal women of African descent.

## Background

Breast cancer has unique racial/ethnic variations in incidence and burden. While the incidence is rising globally, the increase is occurring faster in population groups that hitherto enjoyed low incidence. Reasons for the racial/ethnic variation in breast cancer incidence are unclear; however, differences in the distribution of polymorphisms in key candidate genes involved in estrogen/xenobiotic metabolism might contribute to variation in breast cancer susceptibility in different populations. The *CYP1B1 *gene is located on chromosome 2p21-p22 and contains three exons [1-3]. The entire coding sequence of the gene, however, is contained in exons 2 and 3 [1-3]; exon 3 encodes the heme-binding region of the enzyme [4]. *CYP1B1 *might be involved in hormonal carcinogenesis through its ability to catalyze the metabolism of estradiol to 2-hydroxyestradiol and 4-hydroxyestradiol [5]. The former metabolite is mainly produced in the liver whereas significant amount of 4-hydroxyestradiol are formed in extrahepatic tissues. Whereas 2-hydroxyestradiol has little or no carcinogenic activity, 4-hydroxyestradiol and estrogen have been shown to be potent carcinogens in animal models [6] and humans [7,8]. In-situ conjugation of this metabolite by phase II enzymes is relatively low in extrahepatic tissues, thus leading to its accumulation. The carcinogenic activity of 4-hydroxyestradiol could be due to its hormonal activity, which in some biological assays has been shown to be even higher than the parent hormone. In addition, 4-hydroxyestradiol can undergo redox cycling [7] which results in the formation of free radicals such as superoxide and in the generation of reactive semiquinone/quinone intermediates that have been shown to damage biological target molecules such as DNA [8].

Six polymorphisms of the *CYP1B1 *gene have been described in the Caucasian populations, of which four results in amino acid substitutions [3,4]. Two of these amino acid substitutions are located in exon 3, which encodes the heme-binding domain: codon 432 Val → Leu and 453 Asn → Ser [4] while two other amino acid substitutions are found in codons 48 Arg → Gly and 119 Ala → Ser in exon 2 [3]. The codon 432 Val → Leu polymorphism creates and Eco57I site. Several molecular epidemiological studies have evaluated the association of polymorphisms in CYP1B1 gene and breast cancer risk in various populations, including seven in Caucasian [4,9-14] and four in Asian populations [15-18]. There is scanty data on the role of polymorphisms in this gene in breast cancer susceptibility in populations of African descent. Only one of the US studies recruited a small sample of African-American women; there are no studies on sub-Saharan African populations. Although, several polymorphisms have been described in the *CYP1B1 *gene, we have chosen in this exploratory study to evaluate the role of the *CYP1B1 *Val432Leu polymorphism in breast cancer susceptibility in Nigerian women since there is experimental evidence suggesting that the variant *CYP1B1 *432Leu allele exceeded the wild-type *CYP1B1 *432Val allele with respect to estrogen hydroxylation activities. Given the carcinogenic and estrogenic potential of 4-OH-E2, it is plausible to speculate that inheritance of the *CYP1B1 *432Leu allele may contribute to increased breast cancer risk associated with estrogen-mediated carcinogenesis.

## Materials and methods

### Subjects

Study participants were recruited between September 2002 and April 2004 in four University Teaching Hospitals in Southern Nigeria including University of Benin Teaching Hospital, Benin City; Nnamdi Azikiwe University Teaching Hospital, Nnewi; University of Nigeria Teaching Hospital, Enugu; and University of Port Harcourt Teaching Hospital, Port Harcourt. The Institutional Review Board of University of Pittsburgh and the Ethics and Research Committees of the Nigerian institutions approved the study prior to commencement. A total of 500 study participants comprising 250 women with incident and prevalent breast cancer and 250 age- and institution-matched controls were recruited for the study. Women with confirmed breast cancer were recruited during surgical out-patient clinic visits or in-patient admissions while control subjects with non-malignant surgical diseases such as road traffic accident and other injuries (n = 168), intestinal obstructions (n = 49), appendicitis/pelvic inflammatory disease (26), urolithiasis and urinary tract infections (n = 5) and cholelithiasis (n = 2) were recruited from the same hospitals. Exclusion criteria include non-confirmation of diagnosis of breast cancer, a diagnosis of other malignant diseases, and refusal of donation of blood samples.

Prior to recruitment, participants signed informed consent after detailed explanation of key points of the study including study objectives, risks and benefits, confidentiality and the rights of participants. Interviewer-administered questionnaires were used for data collection; questions were designed to gather demographic history including age, sex, religion, occupation, exposure to chemical fertilizers and pesticides, and rearing of domestic animals. In addition, obstetric and gynecological history including age at menarche, age at first full-term pregnancy, parity, breastfeeding, age at menopause (for postmenopausal subjects), history of use of hormonal contraceptives and hormone replacement therapy and surgical oophorectomy was obtained. Information about lifestyle habits such as cigarette smoking and alcohol consumption was also obtained from study participants. Anthropometric measurements including height, weight, waist and hip circumferences were taken at the end of the interview.

### Sample donation and preparation

Blood samples were collected at the end of the interview; details are reported elsewhere [19,20]; 10 ml of whole blood was collected in one 10 ml K_3_-EDTA vacutainer tube from each of the study participants and stored in ice packs until it was centrifuged within 10 h of collection and buffy coats collected and stored in 3 ml tubes. All the samples were stored at -20°C in the various study sites in Nigeria and later transferred to the Nigerian coordinating center at the University of Benin Teaching Hospital in Polar Pack -20 C ice packs for frozen shipments and later shipped to University of Pittsburgh in dry ice using express services. Samples were stored at -80°C at the University of Pittsburgh until DNA extraction.

DNA extraction was carried out using QIAamp DNA Mini Kits (for buffy coats) and QIAamp DNA Midi Kits (for blood clots) protocols (QIAGEN Inc. Valencia, CA). The extracted DNA was stored at 4°C until used for PCR and RFLP analysis.

### PCR and RFLP analysis

Genomic DNA from the cases and control subjects were analyzed for the presence of the G to C transversion mutation at codon 432 of the CYP1B1 gene by a PCR-based Restriction Fragment Length Polymorphism (RFLP) assay. PCR amplification of a 650 bp fragment of the *CYP1B1 *gene, including part of exon 3 that contains the polymorphism was carried out using forward primer: TCACTTGCTTTTCTCTCTCC and reverse primer: AATTTCAGCTTGCCTCCTG. A 50 μl PCR reaction mixture containing 2 μl of genomic DNA, 5 μl of deoxynucleotide triphosphates, 5 μl each of forward and reverse primers, 5 μl of 10× buffer, 1.5 μl of MgCl_2 _and 0.5 μl of Taq polymerase was placed in a thermalcycler. After denaturing for 10 min at 95°C, the DNA was amplified for 35 cycles at 95°C for 60 s, 58°C for 60 s, and 72°C for 60 s, followed by a 7 min extension at 72°C. A positive control containing genomic DNA and a negative control containing everything except DNA were included in the PCR experiment. Five μl of each PCR product, including the controls, were verified on a 2% agarose gel to ensure that the expected 650 bp product was generated.

Restriction digest for the DNA fragment was carried out using Eco57I restriction enzyme. Fifteen μl of the PCR product was digested for 16 h overnight at 37°C with 1 unit of Eco57I (New England Biolabs). The product of the restriction digest was mixed with 10 μl of loading dye and verified on a 3% agarose gel (with Ethidium bromide) electrophoresis in a 1× Tris-Borate-EDTA buffer at 200 V for 60 min. The presence of a G at position 1294 (*CYP1B1*-codon 432) generated a unique 650 bp fragment, while the 650 bp fragment was divided into unique 340 bp and 310 bp fragments when position 1294 contains a C. The gels were visualized by UV light and the RFLP gel electrophoresis products were read by two independent persons who were unaware of the identities of samples as either cases or controls.

RFLP assays employing Eco57I restriction enzyme were successful in 228 cases and 226 controls, therefore the analysis is restricted to this sample of women.

### Statistical analysis

Statistical analysis was carried out using the Statistical Analysis System (SAS) software (Version 8.0). Conditional logistic regression was used to assess the association between the *CYP1B1 *genotypes and breast cancer risk in the whole sample. Stratified analyses according to menopausal status were carried out. Relevant risk factors that were identified as significant predictors of breast cancer risk were controlled for in the multivariate logistic regression models.

## Results

### Demographic characteristics

Five hundred participants comprising 250 women with breast cancer and 250 age- and institution-matched controls were recruited from four University Teaching Hospitals in Midwestern and Southeastern Nigeria. However, this report is based on data on 228 cases and 226 control subjects in whom RFLP polymorphism assays on *CYP1B1 *gene were successful. Mean age of cases and control subjects were similar (46.3 ± 11.72 years and 47.3 ± 12.14 years respectively). Using univariate logistic regression models, the following variables including family history of breast cancer, education, ever married, age at fullterm pregnancy (FFTP), parity, duration of breastfeeding, abortion, use of hormone contraceptives, waist/hip ratio, and body mass index (BMI) were found to be significant predictors of breast cancer risk. However, only three variables including Family history of breast cancer (OR = 11.17, 95% CI 1.37–91.33), age at first fullterm pregnancy greater than 20 years (OR = 1.32, 95% CI 1.17–3.41) and waist/hip ratio (OR = 1.90, 95% CI 1.18–3.04) were significant predictors of breast cancer risk in the final multiple logistic regression model as shown in Table 1. More details on distribution of anthropometric and reproductive variables and their association with breast cancer are reported elsewhere [21,22].

**Table 1:** Multiple conditional logistic regression comparing cases and controls. Significant demographic predictors of breast cancer risk [Numbers (Percentages (%)], odds ratio (OR), 95% confidence interval (95% CI)

**Variable**		**Cases**	**Controls**	**OR**	**95% CI**
Family history breast Cancer	Yes	14 (6.00)	1 (0.40)	11.17	1.37, 91.33
	No	214 (94.00)	225 (99.60)	1.00	
					
Age at first fullterm Pregnancy (>20 years)	Yes	162 (77.20)	136 (65.07)	1.32	1.17, 3.41
	No	48 (22.8)	73 (34.93)	1.00	
					
Waist/hip ratio (>0.90)	Yes	147 (64.47)	107 (47.35)	1.90	1.18, 3.04
	No	81 (35.53)	119 (52.65)	1.00	

### Allele and genotype frequencies

#### All women

The *CYP1B1 *(Val) allele was less frequent in cases (0.86) compared to control subjects (0.89). The distribution of the *CYP1B1 *genotype is shown in Table 2. The genotype frequencies of the *CYP1B1 *(Val/Val), *CYP1B1 *(Val/Leu) and *CYP1B1 *(Leu/Leu) in the cases were 0.73, 0.25, and 0.02, respectively while the corresponding frequencies in the control subjects were 0.81, 0.17, and 0.02, respectively. The distribution of *CYP1B1 *(Val/Leu) alleles in the control subjects was in Hardy-Weinberg equilibrium, overall and in both premenopausal and postmenopausal women.

**Table 2:** Distribution of Cytochrome *P4501B1 *alleles and genotypes in relation to breast cancer risk

	**Cases**	**Controls**	**OR (95% CI)**	OR (95% CI)*
**All women**	(n = 228)	(n = 226)		
**Allele frequencies**				
*CYP1B1*(Val)	0.86	0.89		
*CYP1B1*(Leu)	0.14	0.11		
				
**Genotype frequencies**				
*CYP1B1 *(Val/Val)	167 (47.85)	182 (52.15)	1.00	
*CYP1B1 *(Val/Leu)	57 (59.38)	39 (40.62)	1.59 (1.01–2.52)	1.57 (0.99–2.51)
*CYP1B1 *(Leu/Leu)	4 (44.44)	5 (55.6)	0.87 (0.23–3.30)	0.94 (0.24–3.64)
				
*CYP1B1 *(Val/Val)	167 (47.85)	182 (52.15)	1.00	
*CYP1B1 *(Val/Leu) + (Leu/Leu)	61 (58.10)	44 (41.90)	1.51 (0.92–2.35)	1.51 (0.96–2.36)
				
**Pre-menopausal women**	(n = 125)	(n = 129)		
**Allele frequencies**				
*CYP1B1*(Val)	0.85	0.92		
*CYP1B1*(Leu)	0.15	0.08		
				
**Genotype frequencies**				
*CYP1B1 *(Val/Val)	91 (45.50)	109 (54.50)	1.00	
*CYP1B1 *(Val/Leu)	30 (62.50)	18 (37.50)	2.00 (1.05–3.81)	1.97 (1.02–3.81)
*CYP1B1 *(Leu/Leu)	4 (66.67)	2 (33.33)	2.40 (0.43–13.38)	2.64 (0.46–15.23)
				
*CYP1B1 *(Val/Val)	91 (45.50)	109 (54.50)	1.00	
*CYP1B1 *(Val/Leu) + (Leu/Leu)	34 (62.96)	20 (37.04)	2.04 (1.10–3.78)	2.04 (1.07–3.81)
				
**Postmenopausal women**	(n = 103)	(n = 97)		
**Allele frequencies**				
*CYP1B1*(Val)	0.87	0.86		
*CYP1B1*(Leu)	0.13	0.14		
				
**Genotype frequencies**				
*CYP1B1 *(Val/Val)	76 (51.00)	73 (49.00)	1.00	
*CYP1B1 *(Val/Leu)	27 (56.30)	21 (43.80)	1.24 (0.64–2.38)	1.20 (0.62–2.34)
*CYP1B1 *(Leu/Leu)	0 (0.00)	3 (100.00)	**	**
				
*CYP1B1 *(Val/Val)	76 (51.00)	73 (49.00)	1.00	
*CYP1B1 *(Val/Leu) + (Leu/Leu)	27 (52.94)	24 (47.06)	1.08 (0.57–2.04)	1.05 (0.55–2.01)

* Adjusted for waist/hip ratio (WHR)

** Odds ratios could not be determined due to missing values in some cells

#### Premenopausal women

Among 142 premenopausal breast cancer cases and 142 premenopausal control women, the RFLP polymorphism assays were successful in 125 cases and 129 controls. The *CYP1B1 *(Val) allele was less frequent among premenopausal cases (0.85) compared to the control subjects (0.92). As shown in Table 2, the *CYP1B1 *(Val/Leu) and CYP1B1 (Leu/Leu) genotypes were more common in cases compared with the control subjects.

#### Postmenopausal women

Of the 108 postmenopausal breast cancer cases and 108 postmenopausal control subjects, the PCR-based RFLP assays were successful in 103 cases and 97 control subjects. The distribution of the *CYP1B1 *(Val) and *CYP1B1 *(Leu) alleles in postmenopausal breast cancer cases and controls were similar. There were slight differences in the frequency of the *CYP1B1 *(Val/Val), *CYP1B1 *(Val/Leu) and *CYP1B1 *(Leu/Leu) genotypes among cases and control subjects, as shown in Table 2. All the three homozygous variants were control subjects.

### CYP1B1 genotypes and breast cancer risk

#### All women

As shown in Table 2, cases were more likely to harbor the Leu allele than the controls. The heterozygous *CYP1B1 *(Val/Leu) genotype was associated with a significant increased risk of breast cancer (Odds ratio [OR] = 1.59, 95% Confidence Interval [CI] 1.01–2.52), compared with the homozygous wild type *CYP1B1 *(Val/Val) genotype. There were very few study subjects with the homozygous variant *CYP1B1 *(Leu/Leu), four among the cases and five among the control subjects; this genotype was not associated with breast cancer (OR = 0.87, 95% CI 0.23–3.30).

Adjustment for waist/hip ratio (WHR) slightly attenuated the risk associated with the *CYP1B1 *1B1 (Val/Leu) genotype in all women (OR = 1.57, 95% CI 0.99–2.51). The risk associated with the *CYP1B1 *(Leu/Leu) (OR = 0.94, 95% CI 0.24–3.64) and the combined *CYP1B1 *(Val/Leu) and CYP1B1 (Leu/Leu) genotypes (OR = 1.51, 95% CI 0.96–2.36) remained essentially unchanged.

#### Premenopausal women

The heterozygous *CYP1B1 *(Val/Leu) genotype was associated with a significantly increased risk of premenopausal breast cancer (OR = 2.00, 95% 1.05–3.81; the risk associated with the homozygous genotype (Leu/Leu) did not reach significance (OR = 2.40, 95% CI 0.43–13.38). The risk of premenopausal breast cancer with the heterozygous *CYP1B1 *(Val/Leu) and homozygous *CYP1B1 *(Leu/Leu) genotypes combined was 2.04 (95% CI 1.10–3.78) (Table 2). The risk of premenopausal breast cancer associated with the *CYP1B1 *genotypes remained essentially unchanged when WHR was included in the model.

#### Postmenopausal women

There was no association between the *CYP1B1 *polymorphism and breast cancer in post menopausal women. Harboring at least one *CYP1B1 *(Leu) allele was not associated with risk of breast cancer in postmenopausal women (OR = 1.08, 95% CI 0.57–2.04). Controlling for WHR did not significantly alter the risk profiles (Table 2).

## Discussion

Breast cancer is a disease with unique phenotypic manifestations in different racial/population groups. It has been hypothesized that these population-specific characteristics of breast cancer may be partly due to differences in genetic susceptibility to the disease arising from variation in the frequency of different polymorphic alleles of key candidate genes involved in estrogen and xenobiotic metabolism in different populations. Our study was designed to evaluate the hypothesis that the CYP1B1 Val432Leu polymorphisms in the *CYP1B1 *gene, a key candidate gene involved in phase I hydroxylation of estrogens (17β-estradiol and estrone) to 4-hydroxy catechols might contribute to breast cancer risk in Nigerian women.

Comparison of our data with reports from other populations indicates wide variation in the distribution of the *CYP1B1 *codon 432 Val → Leu polymorphism across different populations groups. The frequency of the *CYP1B1 *(Val) allele among control subjects in our study (0.89) is closer to the frequency in African-Americans (0.70) [4] but much higher than the figures reported in Caucasians (0.42) [9], Asians in China (0.46) [18], Japan (0.15) [17], and Korea (0.11) [16].

Our results suggest that harboring one *CYP1B1 *(Leu) allele was significantly associated with breast cancer (OR = 1.59, 95% CI 1.01–2.52). Subgroup analysis based on menopausal status showed that the risk conferred by this polymorphism was essentially restricted to premenopausal women in whom the combination of *CYP1B1 *(Val/Leu) and *CYP1B1 *(Leu/Leu) genotypes was associated with over 2-fold increased risk of breast cancer (OR = 2.04, 95% CI 1.10–3.78). This association was not confirmed in postmenopausal women. The associations were not significantly modified by the adjustment of the data for waist/hip ratio (a surrogate measure of etiologically relevant obesity) in either premenopausal or postmenopausal women, despite the finding of preferential 4-hydroxylation of estrogens in obese women on high fat diet [23].

Several other investigators have evaluated the relationship between *CYP1B1 *polymorphisms and breast cancer in different populations. There appear to be no significant overall association between the *CYP1B1 *Val/Leu variant and breast cancer risk in Caucasian populations in a recent meta-analysis [24]. However, a pooled analysis suggests a possible association of both the Val/Leu and Val/Val genotypes with breast cancer in Caucasians but no significant effect was observed in Asians or African-American subjects [24]. Among the seven studies in Caucasians, three reported no association between the *CYP1B1 *Val432Leu polymorphism and breast cancer risk [4,10,14], while three reported a risk effect for the valine allele [9,11,13]; one study reported an inverse association between the valine allele and breast cancer [12]. Statistical significance for the association between breast cancer and the *CYP1B1 *Val/Val polymorphism was reached in two studies [11,13]. In one of these studies, Listgarten et al. [11] found that harboring the heterozygous Val/Leu genotype was associated with a 2.15-fold increased risk of breast cancer (95% CI 1.31–3.52) while the homozygous mutant (Leu/Leu) conferred a 3.30-fold increased risk (95% CI 1.76–6.19). In the second study of 84 cases and 103 controls among the Turkish population, Kobacas et al. [13] reported an overall association between carriers of at least one Val allele and breast cancer risk among women with body mass index (BMI) > 24 kg/m^2 ^(OR = 2.81, 95% CI 1.38–3.74). Although Bailey et al. [4] failed to demonstrate a significant association between the *CYP1B1 *Val432Leu polymorphism and breast cancer risk, they noted that Caucasian patients with the Val/Val genotype had a significantly higher percentage of breast cancer that were positive for estrogen receptors (ERs) or progesterone receptors (PRs), suggesting that this polymorphism may be functionally important for the expression of these steroid receptors.

A small hospital-based case-control study involving 59 African-American women found no statistically significant association between the *CYP1B1 *Val432Leu polymorphism and breast cancer risk [4]. Of three studies in mixed U.S. populations [25-27], one [26] reported no significant association, while two [25,27] reported an inverse association between the Val/Val genotype and breast cancer (OR = 0.4, 95% CI 0.1–1.0 and OR = 0.7, 95% CI 0.6–0.9, respectively) and for the Val/Val and Val/Leu genotypes combined (OR = 0.4, 95% CI 0.1–1.0 and OR = 0.8, 95% CI 0.7–0.9, respectively). Meta-analysis of these studies in African-American and mixed populations showed no overall significant risk associated with the *CYP1B1 *Val432Leu polymorphism in breast cancer susceptibility in these populations [24].

Meta-analysis of studies on the association between *CYP1B1 *Val432Leu polymorphism and breast cancer risk failed to demonstrate an overall significant association in Asian women [24]. Of the four available studies, three [15,16,26] found no significant association. Only one study in the Chinese population [18] reported that, compared with those with the Val/Val genotype, women with the Leu/Leu genotype had a 2.3-fold (95% CI 1.2–4.5) elevated risk of breast cancer after adjusting for confounding variables, the positive association between the Leu/Leu genotype and breast cancer was more pronounced in postmenopausal women (OR = 3.1, 95% CI 1.0–9.1) than in premenopausal women (OR = 1.9, 95% CI 0.8–4.3).

The differences in breast cancer risk associated with the *CYP1B1 *Val432Leu polymorphism in different populations may be due to several reasons including differences in frequency of Val and Leu alleles in different populations, the relatively small sample size of some studies [4,11,13,25,26], as well as differences in study designs.

The mechanisms through which polymorphisms in *CYP1B1 *might influence breast cancer risk are not completely known. *CYP1B1 *is expressed constitutively in extrahepatic tissues including lung and mammary tissue [28]. Although other cytochrome P450 enzymes, such as *CYP1A2 *and *CYP3A4*, are involved in hepatic and extrahepatic estrogen hydroxylation, *CYP1A1 *and *CYP1B1 *display the highest levels of expression in breast tissue [28]; *CYP1B1 *exceeds *CYP1A1 *in its catalytic efficiency as an estradiol (E_2_) hydroxylase, and differs from *CYP1A1 *in its main site of catalysis [5,29,30]. *CYP1B1 *has its primary activity at the C-4 position of estradiol (E_2_), whereas *CYP1A1 *has its primary activity at the C-2 position. These two metabolites differ greatly in their carcinogenicity. Treatment with 4-OH-E_2_, but not 2-OH-E_2_, induced renal cancer in Syrian hamster [7,31]. Analysis of renal DNA demonstrated that 4-OH-E_2 _significantly increased 8-hydroxydeoxyguanosine levels, whereas 2-OH-E_2 _did not cause oxidative DNA damage [32]. Similarly, 4-OH-E_2 _induced DNA single-strand breaks whereas 2-OH-E_2 _had a negligible effect [33]. Comparison of the corresponding catechol estrogen quinones showed that E_2_-3,4-quinone produced two to three orders of magnitude higher levels of depurinating adducts than E_2_-2,3-quinone. Significantly higher 4-OH-E_2_/2-OH-E_2 _ratios were observed in breast tumor tissue than in adjacent normal breast tissue [34]. These findings suggest a causative role of 4-OH catechol estrogens in carcinogenesis and implicate *CYP1B1 *as a key player in the process.

To the best of our knowledge, ours is the largest case-control study on *CYP1B1 *Val432Leu polymorphism and breast cancer risk conducted in African populations.

The epidemiologic literature on determinants of breast cancer in sub-Saharan African populations is scanty despite the evidence that breast cancer is already a public health problem in these developing countries [35,36] and the burden of the disease is likely to increase as women in these populations adopt Western diets and sedentary lifestyles. The use of hospital controls instead of population-based controls might be a source of systematic bias; poor research infrastructure including the absence of a population-based cancer registry and lack of functional communication facilities limited our choice of recruitment for the control subjects. Recruitment of incident and prevalent cases of breast cancer may also be a source of systematic bias as women with rapidly progressive forms of breast cancer may have died early, leaving us with a subpopulation of less aggressive prevalent cases. This is particularly important given the report of an interaction between the *CYP1B1 *Val → Leu polymorphism and hormone receptor status noted in Caucasian women [4]. The observed preponderance of hormone receptor positive tumors in individuals harboring the Val/Val genotype would result in selective survival advantage of this genotype. However, such an interaction if present in our study population would result in underestimation of the breast cancer risk associated with the presence of the *CYP1B1 *(Leu) allele. The non-availability of facilities for hormone receptor assays during the recruitment of study participants in the Nigerian study sites limited our ability to actually evaluate the presence of such interaction if any.

## Conclusion

This study has demonstrated a role for the *CYP1B1 *Val → Leu polymorphism in premenopausal breast cancer risk in Nigerian women. This polymorphism might be mediating its effect through increased metabolic conversion of estradiol, the main estrogen in premenopausal women, to 4-hydroxy estradiol and estrogen quinone and semiquinone intermediates. The results we report in this study need to be considered in conjunction with clinical and epidemiological information. In fact, breast cancer is predominantly a premenopausal disease in Nigerian women [37], with a disproportionately higher prevalence of hormone receptor negative breast cancer, estimated at about 76% [38]. These data, together with the observation of a higher proportion of the *CYP1B1 *(Val) allele among postmenopausal women with hormone receptor positive breast cancer [4], and of an association between Nigerian premenopausal breast cancer and the *CYP1B1 *(Leu) allele suggest that breast cancer in sub-Saharan African populations may have a different etiopathogenesis than in Caucasian or Asian populations. The understanding of differences in estrogen and xenobiotic metabolism resulting from polymorphic variants in key candidate genes, and their interaction with environmental exposure has the potential to considerably improve our ability to characterize individual risk of breast cancer and enhance our ability to design individual and population-specific control and preventive measures.

## List of abbreviations used

*CYP1B1*: Cytochrome *P4501B1 *gene; DNA: Deoxyribonucleic acid; PCR: Polymerase chain reaction; SNP: Single Nucleotide Polymorphism; *CYP1A2*: Cytochrome *P4501A2 *gene; *CYP3A4*: Cytochrome *P4503A4 *gene; E_2_: 17β-estradiol; 2-OH-E_2_: 2-Hydroxy-Estradiol; 2-OH-E_2_: 4-Hydroxy-Estradiol; ER: Estrogen receptor; PR: Progesterone receptor; SAS: Statistical Analysis Systems.

## Competing interests

The authors declare that they have no competing interests.

## Authors' contributions

MNO, CHB, ET, SJG, REF, LHK, participated in conceptualization, design of the study and preparation of manuscript; MNO, ERE, SNCA, JO and EEOU recruited study participants from Nigeria and organized the transfer of biological samples to the University of Pittsburgh; MNO, CHB, ET, SJG and JMZ carried out the genetic analysis and manuscript preparation.
